# Admission criteria for a cardiovascular short stay unit: a retrospective analysis on a pilot unit

**DOI:** 10.1007/s11739-021-02700-4

**Published:** 2021-03-26

**Authors:** Federico Capone, Leonardo Molinari, Marianna Noale, Lorenzo Previato, Sandro Giannini, Gianna Vettore, Fabrizio Fabris, Alois Saller

**Affiliations:** 1 Department of Medicine, University of Padova Medical School, University of Padua, Padua, Italy; 2 Neuroscience Institute, Aging Branch, National Research Council (CNR), Padua, Italy; 3 Department of Urgent and Emergency Care, University of Padova, Padua, Italy

**Keywords:** Acute medical unit, Short stay unit, Admission criteria, Length of stay

## Abstract

Rapid intensive observation (RIO) units have been created to guarantee high standards of care in a sustainable health-care system. Within short stay units (SSUs), which are a subgroup of RIOs, only rapidly manageable patients should be admitted. Physicians are unable to predict the length of stay (LOS) as objective criteria to make such a prediction are missing. A retrospective observational study was carried out to identify the objective criteria for admission within a cardiovascular care-oriented SSU. Over a period of 317 days, 340 patients (age 69.4 ± 14.7 years) were admitted to a pilot SSU within our internal medicine department. The most frequent diagnoses were chest pain (45.9%), syncope (12.9%), and supraventricular arrhythmias (11.2%). The median LOS was 4 days (quartile 1:3; quartile 3:7). Predictors of LOS ≤ 96 h were age < 80, hemoglobin > 115 g/L, estimated glomerular filtration rate > 45 mL/min/1.73 m^2^, Charlson Comorbidity Index < 3, Barthel Index > 40, diagnosis of chest pain, syncope, supraventricular arrhythmias, or acute heart failure. The HEART (history, ECG, age, risk factors, troponin) score was found to be excellent in risk stratification of patients admitted for chest pain. Blood tests and anamnestic variables can be used to predict the LOS and thus SSU admission. The HEART score may help in the classification of patients with chest pain admitted to an SSU.

## Introduction

The management of hospitalized patients is requiring an increasing amount of resources given the impact of the clinical complexities of an aging population on the health-care systems [1, 2]. A growing body of evidence has shown that resource management can be optimized to achieve sustainability while guaranteeing high standards of care by reorganizing the health-care model and services [3]. Rapid intensive observation (RIO) units represent an increasingly applied model as they can guarantee advantages to the patients and the institutions [4], including reducing the length of stay (LOS), facilitating a safe discharge with a lower readmission rate, reducing the 30-day and 1-year mortality [5–7], improving the patient’s satisfaction, and the physician’s quality of work [8].

Among different types of RIOs, one of the most widespread (especially in the UK) is the acute medical units (AMUs) where all kinds of patients coming in from the emergency department (ED) are admitted [3, 9]. For context, an ED transfers only rapidly manageable patients to an RIO and sends those needing longer care directly to the ordinary ward (OW). The former are often called short stay units (SSUs) [10–12].

In 2011, our internal medicine department (IMD) worked in close collaboration with the facility’s ED to institute an RIO aimed to discharge patients within 72 h [13]. The ED physician on the sending end and the RIO physician on the receiving one discuss each case by phone to decide if RIO admission is appropriate. Patients presenting with chest pain, syncope or supraventricular arrhythmias, and other specific diagnoses are eligible for transfer to the RIO. Cardiovascular (CV) diagnosis accounted for approximately 70% of patients sent to the RIO. Approximately 65% of the patients admitted to the AMU for all possible diagnoses were discharged within 72 h (LOS 2.4 ± 0.7 days) [13].

As the organizational model outlined above puts pressure on the ED staff, since they are required to discuss the suitability for OW rather than RIO for each patient, a new model was proposed.

All unselected patients coming from the ED are sent to the IMD. Here, the admitting physician immediately decides whether to admit patients to the RIO rather than to the OW, depending on the available anamnesis and clinical data. How to make this evaluation is crucial; a selection based on the personal assessment of the admitting physician, depending on an empirical LOS prediction, is the most frequently used in SSUs worldwide [12, 14–16]. The physician’s evaluation/intuition cannot be accurate if it is not supported by objective criteria [17–19]. We hypothesize that it is possible to make the LOS estimates based on objective criteria.

Before starting the actual activity of the aforementioned unit, we decided to develop a pilot SSU designed to identify objective criteria that would identify patients eligible for the final SSU. In this pilot unit, patients were hospitalized according to loose criteria to avoid excluding certain categories a priori. A retrospective observational study of the patients admitted to our pilot SSU over a period of 317 days was then conducted. The study aimed to analyze the relationships between the results of the blood tests carried out along with the clinical and the anamnestic variables at admission and clinical outcomes (LOS in particular) in order to fine-tune the entry criteria for SSU admission.

## Patients and methods

A retrospective observational study of patients hospitalized in our conventional IMD and selected for pilot SSU management was conducted. Nine out of the 52 beds of the IMD (the first Clinical Medicine Department of the University of Padova Medical Center in Padova, Italy) were reserved for acute care/observation. Patients were admitted to the pilot SSU by the IMD physicians depending on the anamnestic and clinical data gathered by the ED physicians during their evaluation and consigned to them. Inclusion criteria were as follows: patients with chest pain or showing signs of pathologies such as acute cardiovascular diseases (CVD), cerebrovascular disease, deep vein thrombosis, hypo/hyperglycemia, or presenting signs of onset or worsening of pathologies such as anemia, asthma, electrolyte disorders, or symptoms of undetermined acute inflammatory states or abdominal pain. Exclusion criteria were as follows: age < 18 years and/or the absence of any evident contraindications for a short hospital stay (e.g., signs of a worsening of a physical disability or chronic disease, suspicion of a recurrent or new cancer onset needing further investigation). Three hundred and forty patients were admitted to our SSU during the 317-day observational period (between April 2018 and February 2019). The patients’ mean age was 69.4 ± 14.7 years.

At admission to the IMD, the patient’s medical history was taken, and he/she underwent a thorough physical examination. A blood sample was taken for laboratory testing as ordered by the physician. The laboratory tests considered most important for this phase of the journey were hemoglobin, high-sensitivity cardiac troponin I (Hs-cTnI), and serum creatinine [which the Chronic Kidney Disease Epidemiology Collaboration creatinine equation uses to obtain the estimated glomerular filtration rate (eGFR)]. Hemoglobin and eGFR are known predictors of LOS and in-hospital mortality [20, 21], and troponin levels are measured as means to assess chest pain. Creatinine and hemoglobin levels were analyzed using an automatic analyzer (Technicon Instruments Corp., Tarrytown, NY, USA). The Hs-cTnI assay was carried out using the immunometric method with chemiluminescence detection. The patient’s baseline comorbidity was assessed using the Charlson Comorbidity Index [22], and the Barthel Index [23] was calculated to measure the patient’s level of self-sufficiency. The chest pain score and the HEART score were rated in those patients presenting with chest pain in an attempt to identify the origin of the pain and to stratify the short-term risk of Major Adverse Cardiovascular Events [24, 25]. Given the high incidence of CVD in patients admitted to internal medicine wards worldwide, our SSU is fully equipped with a dedicated cardiovascular diagnostic facility providing cardiovascular services. When necessary, the physicians can perform echocardiography, 24-h Holter monitoring, exercise electrocardiogram (ECG), myocardial perfusion single photon emission computed tomography, which is performed before and after pharmacologic stress (dipyridamole perfusion imaging) or exercise stress (cycle ergometer) in collaboration with the Nuclear Medicine Service.

### Outcome

A LOS of 96 h or less was used as the study’s primary endpoint. A 96- rather than the 72-h threshold was chosen since it was the median LOS in the SSU (see below) and to ensure continuity of care of frail patients admitted to the ward, as transfers from the SSU to the OW were not permitted.

### Statistical analysis

The data were analyzed without imputation of missing values. Categorical variables are presented as numbers and percentages; continuous variables are reported as means and standard deviations (SD) or medians and quartile 1 (Q1) or quartile 3 (Q3). Normal distributions of continuous variables were tested using the Shapiro–Wilk test. The median number of days of hospitalization was computed stratifying the population according to the patients’ characteristics (sex; age group; eGFR; hemoglobin upon admission; Barthel Index upon admission; Charlson Comorbidity Index; admission diagnosis; chest pain score; HEART score; echocardiogram; exercise ECG; known coronary artery disease (CAD); new-onset coronary artery disease (new-onset-CAD) or worsening of a known CAD (worse-CAD); acute coronary syndrome (ACS); housing condition; autonomy; discharge destination). For each characteristic, the median number of days of hospitalization calculated for different strata was compared using the Kruskal–Wallis *H* test or the Wilcoxon rank-sum test.

As specified above, the LOS was dichotomized as ≤ 96 vs. > 96 h. The patients’ characteristics were compared according to dichotomized LOS values, applying Fisher’s exact test or the Chi-squared test for categorical variables; the Wilcoxon rank-sum test was employed to analyze the continuous variables. A multivariable logistic regression model was used to identify the patient characteristics associated with the dichotomized LOS; the covariates considered in the model were those available for the entire sample that were associated with a *p* < 0.20 outcome in the bivariate analyses. The linearity of the covariates was evaluated considering the analysis of the quartiles; possible interactions among the predictors were also assessed. Odds ratios (OR) together with their 95% confidence intervals (95% CI) were estimated.

Other logistic regression models were used for the patients admitted for chest pain and ACS using the new-onset or worse CAD as outcomes. The covariates were identified using a stepwise selection procedure (*p* = 0.20 to entry, *p* = 0.10 to stay).

A *p* value < 0.05 was considered statistically significant. Analyses were performed using SAS 9.4 software (SAS Inc., Cary, NC).

## Results

### Baseline characteristics

The characteristics of the patients admitted to our SSU during the 317-day trial period are summarized in Table 1. Data analysis uncovered that 32% of the patients had anemia (*n* = 109), 28% had an eGFR ≤ 60 mL/min/1.73 m^2^, and 15.6% had values below 45 mL/min/1.73 m^2^. The median value of the Charlson Comorbidity Index was 2 (0–3). Two hundred and ninety-three patients (86.9% of the sample) reported being fully independent at home; the remaining needed some assistance, which was provided by a relative or a professional caregiver. The HEART score was calculated for those patients presenting with chest pain as their primary (*n* = 156, 45.9% of the sample) or secondary symptom (*n* = 24, 7% of the sample), total *n* = 180, 52.9% of the sample.

**Table 1:** Patient characteristics

	Total (*n* = 340)
Sex, females, *n* (%)	154 (45.3)
Age, mean ± SD	69.4 ± 14.7
LOS, days, median (Q1, Q3)	4 (3, 7)
LOS ≤ 72 h, *n* (%)	130 (38.2)
eGFR mL/min/1.73 m^2^, mean ± SD	72.9 ± 26.7
eGFR ≤ 45 mL/min/1.73 m^2^, *n* (%)	53 (15.6)
Hemoglobin upon admission g/L, mean ± SD	132.1 ± 20.6
Hemoglobin upon admission ≤ 130 g/L (males) or ≤ 120 g/L (females), *n* (%)	109 (32.1)
Hs-cTnI upon admission ng/L, median (Q1, Q3) (*n* = 290 patients)	8.0 (3.0, 32.0)
Hs-cTnI upon admission ≥ 34 ng/L males or ≥ 16 ng/L females, *n* (%)	90 (31.0)
Barthel Index upon admission (calculated on in-hospital performance) mean ± SD	63.3 ± 26.4
Barthel Index upon admission (calculated on in-hospital performance), *n* (%)	
Independent (≥ 80)	118 (34.7)
Mild dependency (≥ 60 and < 80)	74 (21.8)
Moderate dependency (≥ 40 and < 60)	100 (29.4)
Severe dependency (≥ 20 and < 40)	31 (9.1)
Total dependency (< 20)	17 (5.0)
Housing situation at the time of admission, *n* (%)	
Living at home	330 (97.6)
Living in a retirement home	4 (1.2)
Living in an extended care unit	3 (0.9)
Hospital	1 (0.3)
Autonomy, *n* (%)	
Fully independent	293 (86.9)
Requires assistance that is provided by a relative	35 (10.4)
Requires assistance that is provided by a professional caregiver	9 (2.7)
Discharge destination, *n* (%)	
Home	330 (97.6)
Retirement home	4 (1.2)
Extended care unit	2 (0.6)
Hospital ward	2 (0.6)
Chest pain score, *n* (%) (*n* = 179 patients)	
< 4	88 (49.2)
≥ 4	91 (50.8)
Heart score, *n* (%) (*n* = 180 patients)	
0–3	49 (27.2)
4	42 (23.3)
5–6	67 (37.2)
7–10	22 (12.2)
Exercise ECG, *n* (%) (*n* = 96 patients)	
Positive	12 (12.5)
Uninterpretable	35 (36.5)
Negative	49 (51.0)
Pre-existing coronary artery disease, *n* (%)	87 (25.6)
New-onset coronary artery disease (new-onset-CAD) or worsening of a known CAD (worse-CAD), *n* (%)	50 (14.7)
Acute coronary syndrome (ACS),* n* (%)	43 (12.9)
30-Day hospital readmission, *n *(%)	33 (9.7)

The most frequent working diagnosis in the patients studied was chest pain, followed by syncope and supraventricular arrhythmias (Table 2). Overall, 227 patients (79.4% of the sample) underwent an echocardiogram; 96 patients (28%) underwent an exercise ECG; 60 patients (17.7%) underwent coronary angiography; and an undiagnosed coronaropathy was found in 40 patients. An ACS was found in 12.9% of the patients. Worse or a new-onset CAD was found in 50 patients (14.7% of the entire sample, 26.9% of the patients admitted for chest pain). The most frequent formulated working diagnoses were chest pain (*n* = 156), syncope (*n* = 44), supraventricular arrhythmia (*n* = 38), acute heart failure (*n* = 29), acute inflammatory states (*n* = 16), and transient ischemic attack (TIA)/minor stroke (*n* = 7).

**Table 2:** Working diagnosis at admission

Diagnosis, *n* (%)	Total (*n* = 340)
Chest pain	156 (45.9)
Syncope	44 (12.9)
Supraventricular arrhythmias	38 (11.2)
TIA/minor stroke	7 (1.2)
Deep vein thrombosis	4 (1.2)
Hypo-/hyperglycemia	1 (0.3)
Anemia	6 (1.8)
Acute heart failure	29 (8.5)
Asthma	2 (0.6)
Electrolyte disorders	4 (1.2)
Acute inflammatory states	16 (4.7)
Abdominal pain	3 (0.9)
Other	30 (8.8)

### LOS

The median LOS of the entire sample studied was 4 days (Q1 = 3, Q3 = 7). The patient characteristics associated with a short LOS according to the analysis were age, the eGFR, hemoglobin levels, the Barthel index, and the Charlson Comorbidity Index upon admission.

As shown in Table 3, the patients whose working diagnosis at admission was chest pain, syncope, or supraventricular arrhythmia had a significantly shorter LOS. No patients had died during the study period.

**Table 3:** Patient characteristics and length of stay (days)

	Length of stay (days)	*p* value
	Median (Q1, Q3)	
Sex		0.076
Females	5 (3, 8)	
Males	4 (3, 7)	
Age		**<** **0.001**
< 65	3 (2, 6)	
65–69	5 (3, 8)	
70–74	4 (3, 8)	
75–79	6 (3, 7)	
80–84	5 (4, 7)	
> 85	6 (3, 10)	
eGFR mL/min/1.73 m^2^		**0.017**
≤ 45	6 (4, 8)	
> 45	4 (3, 7)	
Hemoglobin upon admission g/L		**<** **0.001**
≤ 130 g/L (males) or ≤ 120 g/L (females)	6 (3, 10)	
> 130 g/L (males) or > 120 g/L (females)	4 (3, 6)	
Barthel Index upon admission (calculated on in-hospital performance)		**0.008**
Total dependency (< 20)	6 (4, 10)	
Severe dependency (≥ 20 and < 40)	5 (3, 10)	
Moderate dependency (≥ 40 and < 60)	6 (3, 7)	
Mild dependency (≥ 60 and < 80)	4 (3, 6)	
Independent (≥ 80)	4 (2, 7)	
Charlson Comorbidity Index		**<** **0.001**
0	3 (2, 6)	
1, 2	4 (3, 7)	
3, 4, 5+	6 (4, 9)	
Admission diagnosis		**<** **0.001**
Chest pain	4 (2, 6)	
Syncope	4 (3, 6)	
Supraventricular arrhythmias	4.5 (3, 7)	
Acute heart failure	7 (4, 9)	
Other diagnosis (mentioned above)	6 (3, 11)	
Chest pain score (*n* = 179 patients)		0.7557
< 4	4 (2, 7)	
≥ 4	4 (3, 6)	
Heart score (*n* = 180 patients)		**<** **0.001**
0–3	3 (2, 4)	
4	3 (2, 5)	
5–6	5 (3, 7)	
7–10	7 (4, 9)	
Echocardiogram (*n* = 270 patients)		**<** **0.001**
New-onset regional wall motion abnormalities	7.5 (7, 10.5)	
Uninterpretable (poor acoustic window)	5 (4, 7)	
Regional wall motion preserved	4 (3, 6)	
Exercise ECG (*n* = 96 patients)		**0.015**
Positive	7.5 (3, 8.5)	
Uninterpretable	3 (2, 4)	
Negative	3 (2, 4)	
Known coronary artery disease		**0.011**
Yes	5 (3, 9)	
No	4 (3, 7)	
New-onset coronary artery disease (new-onset-CAD) or worsening of a known CAD (worse-CAD)		**0.001**
Yes	7 (4, 9)	
No	4 (3, 7)	
Acute coronary syndrome (ACS)		**<** **0.001**
Yes	7 (6, 9)	
No	4 (3, 7)	
Housing condition		0.402
Home	4 (3, 7)	
Retirement home, extended care unit, hospital	3.5 (2, 7)	
Autonomy		**<** **0.001**
Fully independent	4 (3, 7)	
Need for assistance, provided by a relative or a professional caregiver	6.5 (4, 11.5)	
Discharge destination		0.756
Home	4 (3, 7)	
Retirement home, extended care unit, hospital	6 (2, 12)	

Multivariate logistic regression, defined using complete data from 336 patients, was used to identify the patient characteristics that were independently associated with a LOS of 96 h or less; age < 80 years, hemoglobin level on admission > 115 g/L, eGFR > 45 mL/min/1.73 m^2^, Charlson Comorbidity Index lower than 3, Barthel Index > 40 and diagnosis of chest pain, syncope, supraventricular arrhythmias or acute heart failure were all characteristics associated with a LOS ≤ 96 h (Table 4). Applying these criteria to the sample, and defining a cutoff point of *c* = 0.50 (i.e., if the estimated probability exceeds *c*, then the derived outcome will be equal to 1; in any other case, it will be equal to 0 [26]); the overall rate of the correct classification was estimated as 62%, with 72% of correct classification of LOS ≤ 96 h and 52% of correct classification for the group with a LOS > 96 h.

**Table 4:** Multiple logistic regression model for “LOS ≤ 96 h vs > 96 h” (data available for 336 patients)

	OR	95% CI	*p* value
Age ≥ 80	0.74	0.41–1.34	0.323
Sex, male vs female	1.41	0.86–2.29	0.171
Hemoglobin levels upon admission ≤ 115 g/L	0.42	0.21–0.84	0.013
eGFR upon admission ≤ 45 mL/min/1.73 m^2^	1.54	0.74–3.22	0.245
Admission diagnosis			
Chest pain vs other	2.11	1.06–4.23	0.034
Syncope vs other	1.24	0.53–2.91	0.622
Supraventricular arrhythmias vs other	1.17	0.48–2.83	0.736
Acute heart failure vs other	0.66	0.23–1.86	0.431
Acute inflammatory states vs other	0.16	0.03–0.82	0.028
Charlson Comorbidity Index upon admission ≥ 3	0.37	0.22–0.64	< 0.001
Barthel Index upon admission < 40	1.72	0.79–3.72	0.169

### 30-Day hospital readmission rate

There was a 9.71% 30-day hospital readmission rate for the entire study group. It was 7.74% (*p* < 0.001, McNemar Test) for patients with higher hemoglobin levels at admission (≥ 110 g/L).

### Chest pain and HEART score

The HEART score value was found to be an excellent tool to stratify the risk of patients admitted for chest pain. A multivariate logistic regression model was used to analyze the participants’ sex, age, troponin values, and HEART score. Using a cutoff point of *c* = 0.50, the overall correct classification rate of healthy patients with new-onset/worse CAD was 80%, with 93% specificity and 40% sensitivity.

None of the patients with a HEART score lower than 4 had ACS, and only 1 had a new-onset-CAD/worse-CAD. Patients with a HEART Score ≥ 7 had an almost fourfold higher risk [OR = 3.83 (95% CI 1.17, 12.5)] of developing new-onset/worse-CAD with respect to patients whose score was ≤ 6.

## Discussion

Hospitals are facing the challenges of evaluating and treating an ever-rising number of patients presenting with a vast range of conditions, pathologies, and, frequently, comorbidities. Counterintuitively, it has been seen that care in SSUs, which are generally instituted within larger traditional internal medicine wards, can improve the outcomes of patients with respect to the conventional hospital model. Some studies have indicated that SSUs can reduce the number of days spent in the hospital, which can be advantageous for a number of reasons, and reduce overcrowding in hospitals and congestion in the ED. As public health officials seek new ways to implement structural reforms to optimize health-care expenditure without compromising the quality of care, the idea of short stay or AMUs is becoming increasingly attractive. In fact, in the UK, the Royal College of Physicians of London has been recommending that these types of units are created to respond to the increasingly complex demands being placed on hospitals.

However, what patients can best be treated in this type of unit, what criteria can be used to select them, and what are the outcomes of these patients are some of the questions that the study set out to answer.

The current study and a previous one [13] were carried out by the same group of investigators who examined two types of SSUs that were organized in slightly different ways in an IMD of a tertiary hospital. During the first trial, the decision of admitting a patient to an RIO or to other destinations was made by the ED physician and one of the specialized physicians working in the unit who exchanged information during telephone conversations. During the second trial, the goal was to create an SSU where patients were hospitalized on the basis of an assessment made by the admitting physician. The problem was to understand on the basis of what criteria the patient was judged suitable or not for admission to the SSU. We therefore developed a pilot unit to define objective criteria that the admitting physician could have applied to admit patients to the SSU.

When the data of the patients studied during the two SSU experiences were analyzed, we saw that the mean LOS of the latest study was notably higher. Firstly, having intentionally chosen loose rather than strict criteria for admission has resulted in the inclusion of a less selected population, which included patients with probably a longer LOS. It is nevertheless true that during the current study, we admitted patients who had worse activities of daily living functioning (demonstrated by the lower Barthel Index scores). Moreover, all patients who were admitted to the SSU were managed there to ensure continuity of care; this policy might have lengthened the LOS.

When the patients’ data were analyzed to verify which characteristics were associated with a shorter LOS, we found that patients admitted with a CVD diagnosis (chest pain, syncope, supraventricular arrhythmias, and acute heart failure) who were fully independent at home, younger than 80 years, and who had the following characteristics upon admission (Charlson Comorbidity Index < 3, Barthel Index > 40, hemoglobin levels > 115 g/L, and eGFR > 45 mL/min/1.73 m^2^) had a shorter LOS. The probability of a LOS ≤ 96 h in patients meeting all of these criteria was higher than 70%. Patients with hemoglobin levels > 110 g/L had also a lower 30-day hospital readmission rate.

As the SSU studied here was cardiovascular care oriented, we were able to carry out a subgroup analysis of the patients admitted because of chest pain. The accuracy of the HEART score used in our patients with chest pain in stratifying the risk of ACS or new-onset/worse CAD was analyzed. Statistical analysis showed that we could immediately exclude the possibility of ACS in those patients with a HEART score < 4 upon admission. It is possible, of course, that these findings would have been altered if our population sample had been larger, but they nonetheless indicate that patients admitted for chest pain with a low HEART score should probably be discharged rapidly instead of undergoing additional diagnostic tests that may be useless (as well as expensive, and at times harmful). Patients with a HEART score higher than (or equal to) 7 should instead undergo an immediate coronary angiography given the high probability of new-onset-CAD/worse-CAD and/or ACS. Using the HEART score in this context could shorten the LOS of some patients and avoid dangerous delays in others. Although the value of these findings is limited by the small population sample studied, these thresholds may be a useful tool for stratifying the risk of patients with chest pain in other SSU settings.

The study’s major limitation is the small population that was studied, while its strengths are the comprehensive nature of the evaluation the patients underwent under the direction of an experienced team of professionals and the use of rigorous statistical methods.

## Conclusions

Objective criteria such as those listed above may be useful to physicians who are evaluating the appropriateness of admitting a patient to an SSU and to predict the LOS. Managing patients presenting to the ED in this manner can both shorten hospital stays and reduce the cost of hospitalization. It can also reduce the risk of medical errors inherent to the strict application of standardized protocols and other hospital-linked complications such as infections. The HEART score is a particularly useful tool in this context for patients with chest pain as it enables physicians to make the decision to discharge them if their score is below 4 or to order additional tests if it is higher than 7 (or 6, in selected cases). Large multicentric controlled studies are warranted to validate these findings.

## Data Availability

The data, analytic methods, and study materials will be available to other researchers for purposes of reproducing the results or replicating the procedure on reasonable request.
